# “It’s Always There, Right?” Exploring Internal Medicine Teams’ Use of Basic Science Knowledge on Inpatient Rounds

**DOI:** 10.5334/pme.1766

**Published:** 2025-06-06

**Authors:** Tracy B. Fulton, John C. Penner, Sally A. Collins, Marieke van der Schaaf, Bridget C. O’Brien

**Affiliations:** 1 Department of Biochemistry and Biophysics, School of Medicine, University of California, San Francisco, CA, US; 2 Department of Medicine, San Francisco VA Medical Center, University of California, San Francisco, CA, US; 3 Center for Faculty Educators, School of Medicine, University of California, San Francisco, CA, US; 4 Director Utrecht Center for Research and Development of Health Professions Education, University Medical Center Utrecht, The Netherlands; 5 Department of Medicine and education scientist, Center for Faculty Educators, School of Medicine, University of California, San Francisco, CA, US

## Abstract

**Purpose:**

Attendings and trainees are expected to use basic science knowledge (BSK) in clinical practice and learning. Evidence of how is scant, and more research situated in clinical learning environments (CLEs) is needed. Our study aimed to characterize use of BSK during patient care in a CLE and the actions, interactions, materials, and beliefs that influence its use, to inform efforts to connect BSK education to clinical workplace learning and patient care.

**Method:**

We conducted a constructivist grounded theory study, collecting data from eight inpatient Internal Medicine (IM) teams at one US institution from October 2022 to January 2023. Data included field notes from 27 hours observing inpatient rounds and 24 team member interviews. Iterative data analysis involved coding, memo writing, and constant comparison to develop a theory of BSK use on IM inpatient rounds.

**Results:**

We found that BSK can be activated on rounds through social interactions (among team members, with patients), and in contact with materials (e.g. the electronic health record). Our findings describe BSK as activated when team members connect to and test BSK or when they explore uncertainty; and that activation is supported by beliefs that BSK use brings value to patient care. However, team members often left BSK not activated, accompanied by beliefs about it not being a good fit for patient care activities. Actions associated with leaving BSK not activated included delegating responsibility for using it, deferring discussion, or curtailing conversations about it.

**Discussion:**

Much of the literature describes BSK use in the clinical setting as the product of individual cognition. Our findings extend this characterization, illustrating how social and material elements of the inpatient CLE influence BSK use on rounds. These elements can be leveraged to activate BSK to support integration of learning with patient care in the workplace.

## Introduction

Basic science, including mechanistic and conceptual principles of biomedical disciplines including anatomy, biochemistry, genetics, pathology, pharmacology, and physiology, is included in medical curricula on the premise that it plays important roles in physician training and practice [1,2,3]. For example, a number of studies show that experienced clinicians’ basic science knowledge (BSK) is activated in clinical reasoning to solve complex problems, and is often used unconsciously during routine practice [4,5,6,7]. Research also suggests BSK becomes ‘encapsulated’ in clinical knowledge over time [8], with physicians reporting varying degrees of BSK use in practice [9,10,11,12,13]. Clinical educators perceive they draw on BSK in teaching, but students’ learning and perceptions about exposure to BSK vary [13,14]. Clinical educators disagree on the amount, type, and form of BSK needed for learning [15,16,17,18,19,20]. Amidst these conflicting views and limited evidence, educators face uncertainty about how to best engage with BSK for optimal clinical learning, teaching, and practice.

Studies suggest that BSK aids learners’ development, retention, and application of clinical knowledge by providing a scaffold for elaborated knowledge structures (i.e. illness scripts) [21,22]. As such, BSK learning during medical school can prepare students to address novel and complex problems [23,24,25], and may contribute positively to identity formation [26,27]. In translating this understanding to curriculum design, medical educators have created programs, courses, and sessions that support cognitive integration of conceptual BSK with clinical applications, aiming to promote connections and transfer of BSK to the clinical workplace [28,29]. However, most studies examining transfer of BSK are conducted under controlled conditions outside the CLE, leaving gaps in understanding of how learners use BSK within clinical workplaces.

In several studies, students report minimal exposure to and use of BSK in the CLE by themselves and by supervisors, and perceive BSK to be taught less than supervisors think it is taught [13,30,31,22,33,34]. Some studies suggest that supervisors’ discomfort with BSK, when combined with a supervisor-dependent model and minimal time for learning on the wards, leaves BSK minimally present [30,32,35]. One observational study reports few instances in which a discussion involving BSK is recognizable during inpatient rounds [35]. These studies present an incomplete picture of how and why BSK is used during patient care, centering students and attendings as responsible for enactment of BSK while paying little attention to other interactions. Our study aimed to construct a theory of BSK use in the CLE. We chose inpatient rounds as the context for our study, given the importance of this setting for teaching and learning, and for making patient care decisions. Our research questions were: (1) How is BSK used by teams in the CLE during inpatient rounds? (2) What actions, interactions, materials, and beliefs contribute to or inhibit its use? A theory of BSK use in the CLE can provide new insights for connecting BSK education to workplace learning and patient care.

## Method

### Design

We designed a constructivist grounded theory (CGT) [36] study to explore our research questions, which are not explained by existing theory. In accordance with CGT, we were sensitized to the philosophical perspective of symbolic interactionism, “…a dynamic perspective that assumes continuous reciprocal processes occurring between the individual, collectivity, and environment” [36]. We aimed to construct concepts grounded in our participants’ experiences by combining non-participant observations and interviews. Given clinical team members might not be aware of or sensitive to BSK use, observations were important to capture actions, interactions, and contextual details that participants might not articulate. Follow-up interviews allowed participants to share their beliefs and reflections and for us to check our understanding of events, yielding multiple views of observed and recollected incidents involving BSK. UCSF Institutional Review Board approved this study as Exempt.

### Setting

We observed and interviewed members of internal medicine (IM) teaching teams at a public US health sciences university. These teams are responsible for care of admitted patients, and have hierarchical yet interdependent roles in teaching, learning, and patient care. Each rounding team had 3–7 members including a faculty clinician (attending), a senior resident, 1–2 1^st^-year residents (interns), 1–2 medical students, and sometimes a pharmacy resident.

### Team composition and reflexivity

Our research team included experts in medicine, health professions education, and educational research. T.B.F. is a basic science medical educator and a preclerkship curriculum leader; J.C.P. is an IM clinician educator. Both have experience with qualitative research, and with creating learning tools designed to link mechanisms to patient care decisions. Other authors are experienced qualitative health professions education researchers. T.B.F. conducted all observations of rounds for consistency and because of her experience with and sensitivity to recognizing basic science discussions in clinical practice. T.B.F. and J.C.P. partnered in data analysis to leverage their insights and perceptions about what constitutes use of basic science, and how to capture authentic interactions during inpatient rounds. We were aware that as a former teacher of many participants T.B.F.’s presence may have influenced participants’ behaviors, perceptions, and what they shared. To mitigate this, before each observation and interview we emphasized that the study aims were not to assess individuals’ or teams’ knowledge, behavior, or skill. Throughout the study authors engaged in reflexive discussions to negotiate different interpretations, co-construct meaning, and address how each author’s background and perspectives shaped the research process, the data captured, and our interpretations [37,38].

### Data collection

From October 2022 to January 2023, we conducted observations of teams and semi-structured interviews with team members. We recruited teams based on availability and student presence to represent perspectives across developmental stages. We included teams that were in each stage of call cycle (on call (admitting new patients), post-call, and pre-call). We interviewed team members individually based on their roles and involvement in key exchanges where BSK appeared or could have appeared. We emailed participants in advance for consent and gave gift cards for participation ($5 for being observed, $25 for interview).

Each team observation lasted 2–4 hours and included all inpatient rounds activities that could be observed by one person, including the team huddle, patient presentations and discussions, and patient interactions. T.B.F. focused on capturing actions and interactions in which a basic science mechanism or concept was suggested or described, which could be probed during interviews. T.B.F. used an EchoII pen (Livescribe Inc.) to record audio and jot notes into a template that highlighted key verbal exchanges, along with information about participants, locations, the physical environment, and nonverbal nuances [39]. The jottings facilitated T.B.F.’s production of field notes from the audio recordings, enriched for descriptions of interactions in context.

We developed a semi-structured interview guide (Appendix A) focused on perceptions of and beliefs about the presence and use of BSK during rounds. We defined basic science for our participants as mechanisms or concepts from biomedical science disciplines that students are exposed to in medical school prior to entering the CLE. We conducted 30-minute interviews individually for each of three participants per team: the attending or senior resident and either two interns or one intern and one student. Audio was collected using the Rev.com app on iPhone. Interviews were conducted either in person on observation days or via Zoom within 24 hours of the observation.

We iteratively modified data collection as we analyzed data and constructed the theory. For example, as T.B.F. and J.C.P. identified moments of BSK use early in data analysis, observations increasingly focused on capturing actions, interactions and materials associated with BSK’s presence and absence, and interviews increasingly focused on participants’ experiences of those events, along with their beliefs and assumptions.

### Data analysis

CGT methodology guided data analysis through iterative coding phases, applied during and after data collection [36]. We focused our analysis on team members’ actions, interactions, and beliefs given our interest in social processes related to BSK use [40]. In initial coding, T.B.F. and J.C.P. used open, line-by-line coding, and identified two broad categories that reflected possible BSK use: 1) explicit discussion of concepts or mechanisms on rounds or recollection during interviews, and 2) lack of explicit discussion or recall despite the potential presence of BSK, signaled via mention of lab results, an algorithm, or allusion to a mechanism. As a group we developed focused codes representing social processes related to “activating” and “not activating” BSK.

**Figure 1:**
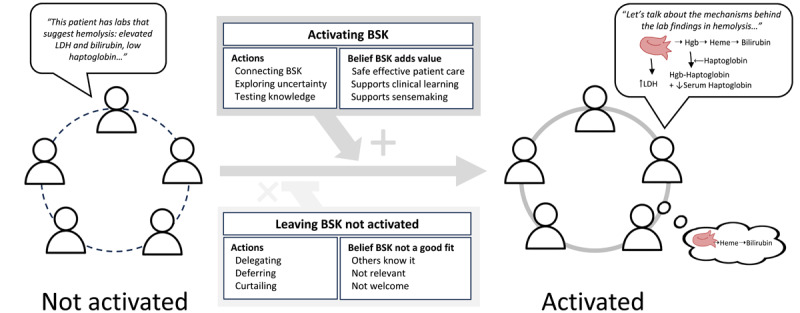
A model illustrating a theory of the use of BSK during inpatient rounds. An example is included here that shows how BSK can be activated or left not activated in the setting of a patient with labs suggesting hemolysis.

T.B.F., J.C.P., and S.A.C. used Dedoose software version 9.0.84 (SocioCultural Research Consultants, LLC) to apply focused codes to all data. All authors created memos from the coded data. We refined our categories and developed conceptual descriptions of their relationships through ongoing comparison of observed and participant-recalled events, and engaged in theoretical sampling by testing our codes, categories, and evolving our theory with new data. T.B.F. collected data from one additional team in May 2023; we gained no further insights regarding categories and concepts, so ended data collection. T.B.F. and J.C.P. created a model (Figure 1), which the research team discussed to ensure it captured the concepts and relationships within the data. Guided by the concepts in our model, T.B.F. generated narratives from field notes and interviews to synthetically and authentically illustrate dynamic interactions between and among individuals on each team and the environment [41]. Data analysis ended when theoretical saturation [36] was achieved and we identified no further insights regarding the theory.

## Results

We analyzed data from 27 hours of observations of inpatient rounds with eight teams (41 team members, 71 patients discussed, 28 of whom were present for at least part of the discussion); and from 24 interviews with students (n = 7), residents (n = 11), and attendings (n = 6) from these teams (Table 1). We constructed a theory of BSK use on rounds that employs concepts of BSK being “activated” and “not activated” (modeled in Figure 1) that are rooted in our participants’ experiences. “Activated” represents the use of BSK in situations in which team members made physiologic mechanisms or other BSK concepts explicit when engaging with others on rounds or with materials in the CLE. “Not activated” represents the presence of BSK when it was not made explicit despite the opportunity. This characterization captures the way that some participants described a perceived presence of BSK on rounds, despite observations and participants’ recollections of its explicit use being uncommon. BSK on rounds was often alluded to in interviews as “under the surface” (A5), “certainly present…not really discussed,” (Ia6) and “running in the background” (MS6).

**Table 1:** Study participants. Observed and interviewed participants, number of patients discussed, and instances of BSK activation per team in this study of BSK use among rounding Internal Medicine teams.


TEAM	TEAM MEMBERS OBSERVED (INTERVIEWED BOLD; PHD ITALICIZED)	TOTAL # TEAM PARTICIPANTS	# PATIENTS DISCUSSED	INSTANCES OF BSK ACTIVATION*

Team 1	**A, Ia, Ib**	3	6	3

Team 2	**A, SR**, Ia, ***Ib***, PR	5	10	5

Team 3	**A**, SR, **Ia**, Ib, **MS**	5	7	5

Team 4	**A**, SR, **Ia**, Ib, **MS**	5	10	2

Team 5	**A**, SR, **Ia**, Ib, ***MS***	5	12	5

Team 6	A, **SR, Ia**, Ib, **MS**	5	10	7

Team 7	**A, SR**, Ia, Ib, **MSa**, MSb, PR	7	8	5

Team 8	A, **SR**, Ia, Ib, **MSa, *MSb***	6	8	3

	***Totals***	***41***	***71***	***35***


*Instances of BSK activation are a tally of the number of times during rounds in which discussion of a BSK mechanism or concept was directly observed or was revealed in an interview as being thought about by the participants. Abbreviations. A: attending; SR: senior resident; I: intern; MS: medical student; PR: pharmacy resident.

The theory describes *activating BSK* and *leaving BSK not activated* as social processes that occur during rounds. These processes are each supported by team members’ actions and beliefs. In a few instances per team observation (Table 1), participants’ actions activated BSK, making it explicit for others and/or themselves. These actions included *connecting* BSK to other forms of knowledge, *exploring uncertainty*, and *testing BSK*. BSK was left not activated when team members’ actions included *deferring* it, *delegating* it to others, or *curtailing* its discussion. Team members’ actions were associated with beliefs related to the *value* or *perceived fit of BSK* for the particular patient care activity or the CLE in general.

Figure 1 illustrates these concepts, which we describe below, and Tables 2 and 3 contain narratives illustrating actions that activate BSK and actions that left it not activated, respectively. Quotes are identified with participants’ roles (A = attending, SR = senior resident, I = intern, MS = student), teams (1–8), and FN if from field notes.

### Activating BSK during inpatient rounds

We identified the process of *activating BSK* in verbal articulations of BSK concepts for the team or a patient, and when individuals described reflecting on it during patient care activities. Such instances of activation were often supported by materials in the CLE, including the electronic health record (EHR) and resources such as websites and textbooks. Although few in number, regular instances of BSK activation occurred with each team (see Table 1), both in the context of conditions common to this service (e.g. hyponatremia, altered liver tests) and in the setting of more complex, rare conditions. We identified three actions associated with BSK activation during rounds: *connecting* BSK to other forms of knowledge, *exploring uncertainty*, and *testing BSK*.

Connecting describes ways in which team members created links between BSK and other forms of knowledge for *other team members, themselves*, and *patients*. Of these instances, it was most common for supervisors to ask questions or make statements intended to link learners’ knowledge about clinical findings to underlying BSK concepts (see Narrative 1 in Table 2). Interactions with materials in the CLE supported team members in connecting clinical decisions to mechanistic justifications. For example, an attending used a chest x-ray from the EHR to discuss the etiology of an opacified lung, integrating concepts of pulmonary anatomy and physiology. Learners described cognitively connecting their own clinical information with memories from study materials, sometimes recalling “vivid” (Ia1) depictions of BSK concepts during patient care interactions, as with an intern whose mental image of the renal tubule informed their understanding of a patient’s diuretic dosing. Finally, multiple interns activated BSK to help patients understand their diagnoses and treatment plans and described needing to “translate it [BSK] into non-medical terms” (Ia6).

**Table 2:** Narratives of activating BSK on rounds.


**Narrative 1: Connecting BSK and other forms of knowledge.** During “sit rounds” Team 1 discusses Patient C, who is undergoing chemotherapy. One intern presents labs showing an elevated white blood cell count. The attending asks, “Thoughts on why the white count is up?” The intern wonders if the chemotherapeutic agents are “lysing cells in some way?” The attending explains “both drugs hit DNA…no way [for them to] cause leukocytosis, rather they cause bone marrow suppression so this has to be something else…What other drugs is he on?” The intern checks the EHR and says, “Oh! A steroid?” The attending asks why a steroid would cause an elevated white count and when the interns cannot remember provides a mechanistic explanation: “It’s a demargination effect. It peels them off the wall.” Both interns nod vigorously and say “Ohhhhhh!” In interviews following rounds, team members confirm this instance of BSK use. The attending identifies these questions as an approach to supporting learners in “***connecting*** it [BSK] to clinical reasoning,” which the attending believes brings value to learning by supporting cognitive integration.” [I] make sure that link to clinical reasoning is there and explicitly stated so that they can really link it back to something that’s practically useful for them.” One intern describes how, during the encounter, they activated knowledge stored in memory as a visual from a textbook studied in medical school. “I honestly couldn’t remember. Um, and then, … [attending] reminded me and it brought me back to those diagrams of like the neutrophils essentially hopping off the wall. That’s why you’re having a rising white count despite not actually having an increase…I think at this point…this is probably gonna be sticking pretty well.”

**Narrative 2. Exploring uncertainty**. During pre-rounds and patient rounds, team 7 discusses Patient S, who has an unusual pattern of abnormal liver tests. The attending tells the team: “This is mostly cholestatic injury. But we see she also has transaminase elevation and normal INR. Could spend more time looking at her liver function tests because they are very very fascinating. Just so everyone is clear, no one knows what this is, this is very weird…” Team members are ***exploring uncertainty*** throughout the morning as they discuss mechanisms that could explain the test results, consulting with hepatology, rheumatology, and pathology. At the end of rounds, a pathologist discusses a liver biopsy slide with the team and diagnoses vanishing bile duct syndrome, which prompts team members to smile and exclaim “wow” and “cool!” In interviews, the attending and senior resident reflected on activating and enhancing their BSK during discussions. The senior resident considered how to “…make sense of all of that, particularly with her intact synthetic function… there’s a lot of like, how does the liver make bile and…how does, which parts of the liver are getting injured and why?” The student described how BSK felt ubiquitous (“…I actually think it was pretty much everything”) but found it challenging to follow the conversation about the complex and rare condition: “I was just looking up all the different terms that were coming up.”

**Narrative 3. Testing BSK knowledge**. Team 3 discusses Patient T, who had a seizure and developed hyponatremia. The MS3 presents the case to the team in a meeting room, saying “I read on [a US website for clinical guidelines] we can enhance effectiveness of salt tablets…by adding a loop diuretic. That’s especially effective for patients with high urine osmolality and it’s around double the serum osms.” The intern thanks the student and notes that for this elderly patient, more aggressive diuresis would not be appropriate given it exacerbates fall risk. The senior resident agrees. In interviews, team members describe this as a moment of ***testing knowledge***. The student reflects, “…I had to like get my bearings on which transport is doing what, what the urine osmolarity means versus the urine sodium; And what, which direction we can expect that to change. It all had to make sense to me internally before I can comfortably pitch it.” The intern reflects that it made sense for the student to propose a treatment based on “first principles” without yet understanding clinical management. The intern themselves also identifies a knowledge gap, wanting to “…go back and kind of look at that Lasix recommendation and make sure that I’m understanding it correctly because I feel very uneasy when I will like dismiss something based on…shaky knowledge…I wanna make sure that that’s correct. Mainly because A, I wanna make sure that I’m doing what’s right for the patient and B, I don’t wanna be teaching something that’s wrong to somebody.”


BSK activation also occurred when team members, especially attendings and senior residents, were *exploring uncertainty* in discussions of complex cases (Narrative 2, Table 2). Participants described BSK as supporting clinical reasoning when patients don’t “follow the algorithm.” (MS3) These explorations were critical in the context of resolving *“*diagnostic dilemma(s)” (A5), but were challenging to follow and understand for early learners.

*Testing BSK*, the least commonly observed approach to BSK activation, refers to situations in which learners verbally attempted to link their own conceptual knowledge from the classroom to patient care during rounds (Narrative 3 in Table 2). Students who attempted to test and transform their classroom knowledge noted that theoretical knowledge does not necessarily translate into practical knowledge.

Many participants, regardless of role, noted circumstances when deliberately activating BSK was important because they believe it *adds value* to workplace learning and practice. They described it adding value through *enabling provision of “safe [patient] care*” (Ia3), *supporting clinical learning*, or through *sensemaking*. Table 4 includes quotes that highlight these beliefs, as do narratives in Table 2.

### Leaving BSK not activated

It was common for BSK to not be activated during patient care activities on rounds. We observed three actions that left it not activated during rounds: *delegating, deferring*, and *curtailing*.

*Delegating* refers to instances when clinical team members indicated that people outside of the team, most often a pharmacy practitioner or a consultant, would apply a deeper understanding of BSK important to the care of the patient being discussed. As such, the team did not need to “dig further” (Ia5) to discuss it (see Narrative 1 in Table 3).

**Table 3:** Narratives of leaving BSK not activated on rounds.


**Narrative 1: Delegating responsibility to use or know BSK to someone else.** Team 6 begins table rounds discussing Patient W, who has respiratory failure and COVID-19. The patient is not sleeping well despite taking a sleep medication. The senior resident questions whether to continue the drug due to its addictive potential. The attending notes that sleep specialists recommended it, leading to a discussion about alternative options. In interview, the senior resident describes relying on the sleep consult notes in the EHR, which include mechanisms of action and justification of the decision. When asked why drug mechanisms were not discussed during rounds, the senior resident describes, “We rely on each other’s notes so much and so…I think for me, I wouldn’t probably go back down a rabbit hole, like a basic science question for that. I would probably just read the sleep specialist notes…usually you can rely on the expertise of your colleagues.” By entrusting their consultant colleague to activate BSK that responsibility was *delegated* outside the team.

**Narrative 2: Deferring BSK to a time outside of rounds**. Team 5 discusses Patient E, who has recently been diagnosed with pancreatic cancer and developed complications of pancreatitis leading to multiple sepsis admissions. While reviewing the patient’s EHR, the attending notes the patient’s rising eosinophil count and says, “From an educational perspective we can talk this afternoon about his rising eosinophilia. When you think about eosinophilia, is this something emergent I need to work on?” The attending lists possible causes (“…adrenal insufficiency, critical illness, drug-related…”) but doesn’t delve into concepts that underlie the framework. In interview, the attending notes that they typically plan such teaching points in advance but must restrict these discussions to a “two minute thing…if the senior resident kind of offers the opportunity.” By scheduling a longer discussion for afternoon teaching, the attending ***deferred*** the activation of BSK until after rounds.

**Narrative 3. Curtailing discussions of BSK**. Team 2 discusses Patient G in the hallway. The team is discussing their ninth patient on-the-go while visiting a select number of patients. The intern presents an 85-year-old patient admitted for COVID pneumonia, with a slightly elevated bilirubin, which is trending down. The senior resident asks: “Think she has Gilbert’s?” The intern replies, “Isn’t it X-linked?” revealing a misunderstanding about the disorder. The team quickly moves past it as the senior resident asks more about the patient’s cough, the attending discusses length of stay for COVID-positive patients and then gives the intern feedback on the presentation. By not taking up a question about a mechanism, team members ***curtailed*** a discussion in which BSK activation might occur.


**Table 4:** Beliefs of BSK use adding value, associated with activating BSK; and beliefs BSK is not a good fit in the CLE, associated with leaving it not activated on rounds.


BSK USE ADDS VALUE BECAUSE IT:

**Enables provision of safe, effective patient care** “I think for every new patient it’s super important to do that…basic science understanding and thinking to dig into what potentially is an alternative pathophysiology for this presentation…that stepping back is really important.” (Ia5) “…basic science is key to clinical excellence because it…gives you this basic kind of basement bottom for which…helps you decide… what’s the most important thing for me to act on today?” (SR6) **Supports clinical learning** “…when you’ve just been, you know, with the books and when you’re jumping into clinical medicine,…that transition is, is very bizarre sometimes. So it helps to tell them about what they already know. [It] makes them comfortable.” (A3) “I think that like most of the time if I like have a basic science explanation for something that’s like not too hard to understand, it does make things like stick in my brain a little bit better.” (Ib2) **Supports sensemaking** “I just really love physiology and I really love thinking about…how the body works. And it’s like a constant source of joy and fascination for me. And I wanna instill that love of learning into our learners because it’s such a privilege to be able to do this work.” (A7) “Partly why I teach that is I try to link it to the evidence…for why we do what we do. So it’s not just like we just do it. Yeah. Part of that is…my intellectual curiosity and trying to draw on intellectual curiosity.” (A2)

BSK USE IS NOT A GOOD FIT BECAUSE:

**It is not relevant** “It’s not that often that I, as a clinician [am] hearkening back to basic science principles to then make clinical decisions.” (A2) “I think…in discussions with patients, we…try to keep basic science on the other part of our brain. Yeah…actually, at the start of rotations, that was a challenge for me.” (MS3) **It is not welcome** “…basic science is really important, but knowing when to evoke it…is sort of like reading the room…a lot of people, talking to classmates, really hate basic science…. Just wanna see cause and effect, like start medication and [see the] clinical effect.” (MS6) “…if I had a more fundamental basic science question, I would usually aim that at the attending, not cause the intern doesn’t have that knowledge, just cause the intern’s so busy…If I’m talking to the intern, I want to have a clinically relevant question that needs answering…” (MS3) **Others know it** “…what I was thinking of was, you know, the sodium/potassium/chloride cotransporter and what is moving with the furosemide or not. I know obviously the attendings and the residents have that knowledge, but…it wasn’t explicitly discussed.” (MS3) “I assume my students have some familiarity… that’s what I’m hoping if something isn’t explicitly discussed.” (A4)


Abbreviations: Participants are identified by role (A = attending, SR = senior resident, I = intern (a or b when 2 were present), MS = student), and team number (1–8). FN = field notes.

Attendings and senior residents *deferred* BSK points to informal afternoon teaching (Narrative 2, Table 3); students and interns *deferred* BSK to time outside of rounds to “go back” (MSa7) and fill knowledge gaps, using a variety of material resources (e.g. flashcards, web pages, clinical manuals that contain “a lot of basic science” (Ia3)).

We identified *curtailing* when students or interns asked questions or made confused statements about BSK concepts that were not taken up by the team (Narrative 3, Table 3), and when team leaders decided that thinking more extensively about a mechanism was not urgent.

The time-limited and pressured nature of inpatient rounds was a key influence on these actions. Despite some participants aspiring to integrate BSK into clinical learning and practice, many associated BSK discussions with “sitting” or “slowing down” (A3, MS4, MSb8), neither of which are often possible during rounds. One participant described the challenge: “I don’t wanna say it’s not practical, but it’s hard. I’m working on shortening rounds as it is… While still making sure the patient feels heard.” (SR2)

As described above, some participants recognized that BSK activation can bring value to inpatient rounds discussions. However, decisions were frequently made that left it not activated given the busyness of rounds and/or the complexity of the patients being discussed. Team members articulated that, in these instances, BSK discussions were not a good fit for the patient care activities at hand. Beliefs that were associated with BSK being left not activated included: it is *not relevant* to the patient’s care urgently, it is *not welcome* (because it is not liked or because team members are too busy), and *others know it* already (Table 4). There were many ways the belief that *others know it* appeared. Attendings and residents articulated the assumption that students understand the BSK concepts relevant to patient care and are already practicing application on rounds silently, or outside of rounds. Students and residents expressed the assumption that supervisors have already integrated BSK into their knowledge structures and apply it to patient care. One intern said, “…it’s always there, right? They’re thinking of it… it’s already woven into their clinical decision making” (Ia4). Quotes that support these beliefs are included in Table 4. The narratives in Table 3 also illustrate some of these beliefs.

## Discussion

We offer a theory of BSK use on patient rounds grounded in the reality of the clinical learning environment. Our findings describe activation of BSK and leaving it not activated as social processes supported by team members’ actions and beliefs, and in some cases by material elements of the CLE. By examining BSK use within the complexities and constraints of the CLE and highlighting social and material influences, this study expands beyond a view of BSK that depends predominantly on it being packaged into cognitive schema, waiting to be extracted by an individual facing a clinical puzzle [28,42,43]. Rather, BSK use can be viewed as “activatable” through interactions with BSK-attuned team members, with extended team members, such as pharmacists or consultants, and even patients during patient care activities [44]. References and material resources such as the EHR or images can further support access to BSK. The theory adds novel insights about where, when, and how BSK is being used, and how, even in circumstances in which activation might only take a few moments, it is often *not* discussed. The theory also highlights ways in which BSK use can, in appropriate circumstances, be socially and materially integrated into patient care activities in ways that clinical team members may not have been aware of, and that may add value.

Our observations of attendings connecting BSK to clinical knowledge echo Pai et al’s observations that attendings drove incorporation of BSK into rounds by engaging learners and sharing “pearls” [35]. We observed several novel circumstances of BSK activation that have not been reported in the literature, all of which are likely to support effective workplace learning. Instances of learners testing their BSK during patient presentations are likely to be an important part of the process by which learners transform prior declarative knowledge to procedural knowledge [45]. The observed activation of BSK as teams explored uncertainty about complex patient cases would seem to position BSK to support diagnosis in non-routine and complex cases [46]. Finally, we also observed team members using BSK concepts to help patients understand their conditions, which is likely to not only support patient satisfaction, but also has been demonstrated to improve understanding and retention of concepts [47].

Despite the observed instances of BSK activation associated with important learning and patient care opportunities, some participants described BSK as rarely essential for routine care or clinical learning. While other studies have suggested that attending discomfort with BSK is a barrier to its use in practice [30,31,32,35], this seemed to be less a concern for most attendings in our study than was the overall complexity of balancing patient care and learning tasks. This combined with participants’ association of BSK activation with slowing down helps explain why it may be deprioritized or overlooked when patient care demands are high and time for teaching is limited, which was common on rounds in our study.

Though participants often assumed that others on the team “know” BSK, it is unclear the extent to which that is accurate. Thus, an environment in which BSK is not activated, or one with instances of BSK activation that do not provide opportunities for feedback has important implications for learning, particularly for students. Such an environment risks leaving encapsulation of this knowledge to chance, as students may not naturally make robust or accurate connections between BSK and their clinical experiences without practice and pedagogical support [33], and may therefore struggle in transforming BSK knowledge for clinical practice [48]. Without exposure to mechanisms during knowledge encapsulation, learners may develop less rich, coherent, stable mental representations, which could have downstream consequences to their clinical reasoning skills [8,49].

Until there is a better understanding of when and how BSK *needs* to be activated to best support learning and patient care, we suggest bringing awareness to potential moments of activation. While we are wary of forcing BSK into an already complex learning environment, and realize that doing so risks cognitive overload and negatively affecting learner identity formation [50], we encourage educators to consider whether options besides curtailing BSK discussions are feasible, even if it means delegating or deferring a BSK discussion away from rounds. Here we suggest such options for attendings, interns, and curriculum designers.

To support BSK activation, attendings can incorporate BSK into teaching scripts [51], which may improve their comfort with BSK if this is an issue, and allow them to deliberately make this thinking visible to learners. This cognitive apprenticeship approach has been suggested to facilitate students’ cognitive integration of basic and clinical science [32,52]. Perhaps more importantly, integration of BSK into scripts for routine clinical problems (e.g., renal physiology in hyponatremia, liver biochemistry in liver function test changes, antibiotic or chemotherapy mechanisms as they relate to features of specific microbiologic organisms or cancers) could increase awareness of these connections and may help overcome students’ tendency to associate BSK mainly with uncommon conditions [33].

Many interns expressed aspirations to connect BSK to their own learning more often and described feeling responsible for shepherding students through clinical learning. However, students expressed hesitation to ask BSK questions of interns. We encourage attendings to explore opportunities for interns to engage with BSK and model its use. For example, attendings might defer a discussion about BSK and delegate it to interns to teaching sessions that take place outside of rounds. This could support their learning and development as clinical teachers, a role that will become increasingly prominent as their training progresses [53,54]. Interns themselves could be encouraged to build either illness or teaching scripts that contain BSK explanations.

Despite its utility, reliance on attending- or intern-driven transmission confines BSK discussion to their knowledge areas, limiting opportunities to “see” what learners see. Curriculum designers could build situated activities into rotations that could support students in integrating BSK with clinical practice. Given the challenges learners described in fully understanding the connections between BSK and practice during moments of activation, it is clear that activation does not necessarily ensure understanding, and that time and explicit pedagogical support from supervisors may be critical. For example, encouraging students to explain BSK concepts to patients or summarizing for the team information from a consult note or conversation could be useful activities that draw on authentic social and material interactions in the CLE. These activities would also demonstrate practical applicability of BSK during patient care [18]. Reflection activities that ask students to describe learning objectives that have facilitated their application of BSK use with patients appear to be a valuable exercise [26,33].

### Limitations

Limitations in our study include having a single observer and interviewer, which limited the number of interviews and team members’ perspectives included for each team. While a single observer-interviewer who many participants knew as a supportive and nonjudgmental teacher was intended to encourage open discussions, it may have inadvertently influenced participants to ascribe value to BSK. We sampled from internal medicine, and our findings may not apply to other specialties. We did not assess the learning experience for individuals during BSK activation, and future studies could examine associations between activation and the quality of learning or the relationship between activation and clinical reasoning.

### Conclusion

By expanding the view of BSK use beyond the minds of single individuals, our theory offers novel perspectives on workplace teaching and learning. The theory provides rich context for why and how BSK is often not discussed as a team during patient care, while illuminating valuable opportunities to support social and material integration of BSK into patient care activities by clinical teams to enhance learning and practice. We encourage educators, leaders, and learners to examine and question their beliefs about BSK’s fit on rounds, and researchers to examine where and when it is most critical that it be activated for optimum learning and patient care.

## Appendix 1

### Interview guide

Thank you so much for sharing your perspective and your time. Your participation today indicates your informed consent. With your permission, I’ll record this interview. Your participation is voluntary so you may ask to skip any questions or stop the interview at any time. All data we collect will be kept confidential and not shared with anyone outside of the research team. Identifying information will be removed from transcripts and audio recordings will be deleted after data analysis. Your participation will not affect your education or future status at UCSF. If you have any questions about the study, you may follow up with me (see Information Sheet that was emailed to you). If you have any questions about your rights as a research subject, you may call the UCSF Institutional Review Board at xx. Do you have any questions before I begin?

I’ll turn on the recorder now. [begin recording]. This is an interview with [participant number] on [date].

### Part I: Information about today’s interactions

1. *Added after teams 1 + 2*: I’m looking for the form basic science knowledge takes in the CLE. So I’d like to start by asking … how do you define basic science knowledge?
2. If they ask, I could share mine… “I’m talking about mechanisms or concepts from biomedical science disciplines that students are exposed to in medical school prior to entering CLE (incl. biochem, physio, genetics, anatomy, pharm, path, etc).”
3. How would you describe the degree to which basic science knowledge was present during rounds today?
4. Did the “amount” of basic science knowledge that came up during rounds today feel typical?
5. Can you tell me a bit more about the instances and ways basic science knowledge was present and the form it took?
  - Probes *(evolved over time):* What form did it take? Where was it? Why was it present/needed? (E.g. Difficulty of case? Interest? Pt interactions?) Where did it or will it go? (For students: Did you do anything with these ‘glimpses’ of BS later in the day or do you plan to?) How was it present for you vs rest of team?
  - Probe: Follow up on where I saw “glimpses” – ie. mentions of concepts, mechanisms, evidence, etc.
  OR – Can you tell me a bit more about why it didn’t come up?
  - Probe: Does that mean it wasn’t present?
  - Probe: Or, does it mean it wasn’t needed?
6. What was your role in the exchange(s) that involved basic science?
  - Probe: How do you see your role generally on the team during rounds?

### Part II: General questions about perceptions about basic science

1. What role do you think basic science knowledge (within the team) plays in patient care?
2. What role do you think basic science knowledge plays in student learning on the wards?
3. (Evolved over time) How do you see your role in these processes? How do you prepare for rounds? Is preparation to discuss basic science with students a part of your preparation?
